# Evaluation of ventricular pacing suppression algorithms in dual chamber pacemaker: Results of “LEADER” study

**DOI:** 10.1002/joa3.13117

**Published:** 2024-07-16

**Authors:** Jongmin Hwang, Seongwook Han, Hyoung‐Seob Park, Tae‐Wan Chung, Minsu Jung, Seung‐Jung Park, Chan‐Hee Lee, Jin Hee Ahn, Eue‐Keun Choi, Myung Hwan Bae, Young Soo Lee, Sang Won Park, Dae In Lee, Yoo‐Ri Kim, Min‐Soo Ahn, Jaemin Shim

**Affiliations:** ^1^ Division of Cardiology, Department of Internal Medicine Keimyung University Dongsan Hospital Daegu Republic of Korea; ^2^ Division of Cardiology, Department of Internal Medicine Heart Vascular and Stroke Institute, Samsung Medical Center, Sungkyunkwan University School of Medicine Seoul Republic of Korea; ^3^ Division of Cardiology, Department of Internal Medicine Yeungnam University Medical Center Daegu Republic of Korea; ^4^ Division of Cardiology, Department of Internal Medicine Pusan National University Hospital Pusan Republic of Korea; ^5^ Division of Cardiology, Department of Internal Medicine Seoul National University Hospital Seoul Republic of Korea; ^6^ Division of Cardiology, Department of Internal Medicine Kyungpook National University Hospital Daegu Republic of Korea; ^7^ Division of Cardiology, Department of Internal Medicine Daegu Catholic University Medical Center Daegu Republic of Korea; ^8^ Cardiovascular Center Bucheon Sejong Hospital Bucheon‐si Republic of Korea; ^9^ Department of Cardiology, Department of Internal Medicine Korea University Guro Hospital Seoul Republic of Korea; ^10^ Division of Cardiology, Department of Internal Medicine Chonnam National University Medical School Gwangju Republic of Korea; ^11^ Division of Cardiology, Department of Internal Medicine Yonsei University Wonju College of Medicine Wonju Republic of Korea; ^12^ Division of Cardiology, Department of Internal Medicine Korea University Medical Center Seoul Republic of Korea

**Keywords:** atrioventricular hysteresis, fixed AV delay, mode‐switch algorithm, sinus node dysfunction, unnecessary right ventricular pacing

## Abstract

**Background:**

There is limited research on the intra‐individual efficacy of ventricular pacing minimization algorithms developed by Biotronik—the Ventricular Pace Suppression algorithm (VpS) and the Intrinsic Rhythm Support plus algorithm (IRSplus) (BIOTRONIK SE & Co. KG, Berlin, Germany). We performed a randomized pilot trial that evaluated the efficacy of two algorithms in patients with symptomatic sinus node dysfunction (SND) who received a dual‐chamber pacemaker.

**Methods:**

The trial was conducted in 11 tertiary hospitals in South Korea. The patients were randomized to either the VpS or IRSplus algorithm group after a 3‐month period of fixed atrioventricular (AV) delay. The primary outcome was the ventricular pacing percentage (Vp%) at each follow‐up visit. The secondary outcomes were the occurrence of heart failure (HF) and atrial fibrillation (AF) during the study period.

**Results:**

Data from 131 patients were analyzed. Initially, their average Vp% over 3 months with a fixed AV interval was 14.1 ± 19.4%. Patients were randomly assigned to VpS and IRSplus groups, with 66 and 65 in each. Algorithms reduced average Vp% to 4.0 ± 11.3% at 9 months and 6.7 ± 14.9% at 15 months. These algorithms were more effective for patients with paced AV delay (PAVD) ≤300 ms compared to those with PAVD >300 ms. Both algorithms were equally effective in reducing Vp%. Clinical AF or HF hospitalization was not observed during the study period.

**Conclusion:**

The VpS and IRSplus algorithms are effective and safe in minimizing unnecessary ventricular pacing in patients with SND.

## Graphical abstract

The VpS and IRSplus algorithms are effective and safe in minimizing unnecessary ventricular pacing in sinus node dysfunction patients with dual chamber pacemakers.

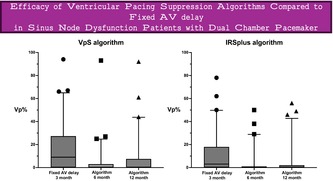

## 1 INTRODUCTION

In the MOST (Mode Selection Trial in Sinus‐Node Dysfunction) trial, dual‐chamber pacing reduces the risk of atrial fibrillation (AF), reduces signs and symptoms of heart failure (HF), and slightly improves the quality of life.^1^ The DANPACE (Dual Chamber Pacing in Sick Sinus Syndrome) trial highlighted a critical concern with single‐lead atrial pacing (AAIR), noting a two‐fold increase in the likelihood of reoperation due to the emergence of atrioventricular (AV) block, which developed at a rate of 0.6%–1.9% per annum among patients.^2^ Based on these findings, dual‐chamber pacing, specifically DDD(R) mode, is a preferred pacing strategy for patients who need a permanent pacemaker (PPM) due to symptomatic SND.^3^ Hence, patients with SND who receive a dual‐chamber PPM may experience inadvertent RV pacing. However, many studies revealed that frequent right ventricular (RV) pacing exceeding 20%–40% can be associated with an increased risk of adverse clinical outcomes, including AF, HF, and even mortality.^4^, ^5^ Therefore, it is essential to minimize unnecessary RV pacing in these groups of patients. Currently, there are three methods: (1) DDD(R) mode with fixed AV delay (the AV delay is fixed to be longer than the patient's intrinsic PR interval); (2) algorithms operating in ADI(R) mode, with a rapid mode switch to DDD(R) mode in case of loss of AV conduction (ADI‐DDD); and (3) algorithms allowing extended AV delays using AV hysteresis (AVH).^6^

Recent research in this domain has been limited, particularly in the algorithms developed by Biotronik—the Ventricular pace Suppression algorithm (VpS, based on ADI‐DDD mode switch) and the Intrinsic Rhythm Support (IRS) plus algorithm (IRSplus, based on AV hysteresis; BIOTRONIK SE & Co. KG, Berlin, Germany). In this study, we conducted a randomized pilot trial to evaluate the efficacy of these ventricular pacing minimization algorithms compared to the fixed AV delay method.

## 2 MATERIALS AND METHODS

This was a multicenter, randomized, single‐blinded, parallel‐group trial conducted in 11 tertiary hospitals in South Korea. Data collection adhered to the principles outlined in the Declaration of Helsinki (2013) and Good Clinical Practices. The study protocol was approved by the Research Ethics Committee of Keimyung University Dongsan Medical Center (DSMC 2018‐08‐0222). Then, the ethics committees and corresponding health authorities of all study participant sites approved the protocol. All the patients provided written informed consent before enrollment. This trial was registered with ClinicalTrials.gov, number NCT 03843242 (LEADER [EvaLuation of ventricular pacing suppression Algorithms in Dual chamber pacEmakeR] study).

### 2.1 Study patients

Patients were recruited from May 2019 to April 2021. Individuals who met the following criteria were enrolled in the study: (1) patients aged 20 or older but less than 85 years who understood the research protocol and had completed the written informed consent form; (2) patients with symptomatic SND confirmed after discontinuation of beta‐blockers, calcium channel blockers, and other drugs that could affect the conduction system; (3) received new implantation of Enitra 8 DR‐T® dual chamber PPM equipped with the IRSplus algorithm and VpS algorithms (BIOTRONIK SE & Co. KG, Berlin, Germany); (4) PR interval <350 ms on 12‐lead electrocardiogram; (5) no evidence of second or third degree AV block; and (6) no previous history of AF. We excluded patients from enrollment if they had undergone generator replacement, if they had a life expectancy of <12 months, if they were pregnant or mentally disabled, or if they would likely be unavailable for follow‐up.

### 2.2 Study design

**FIGURE 1:**
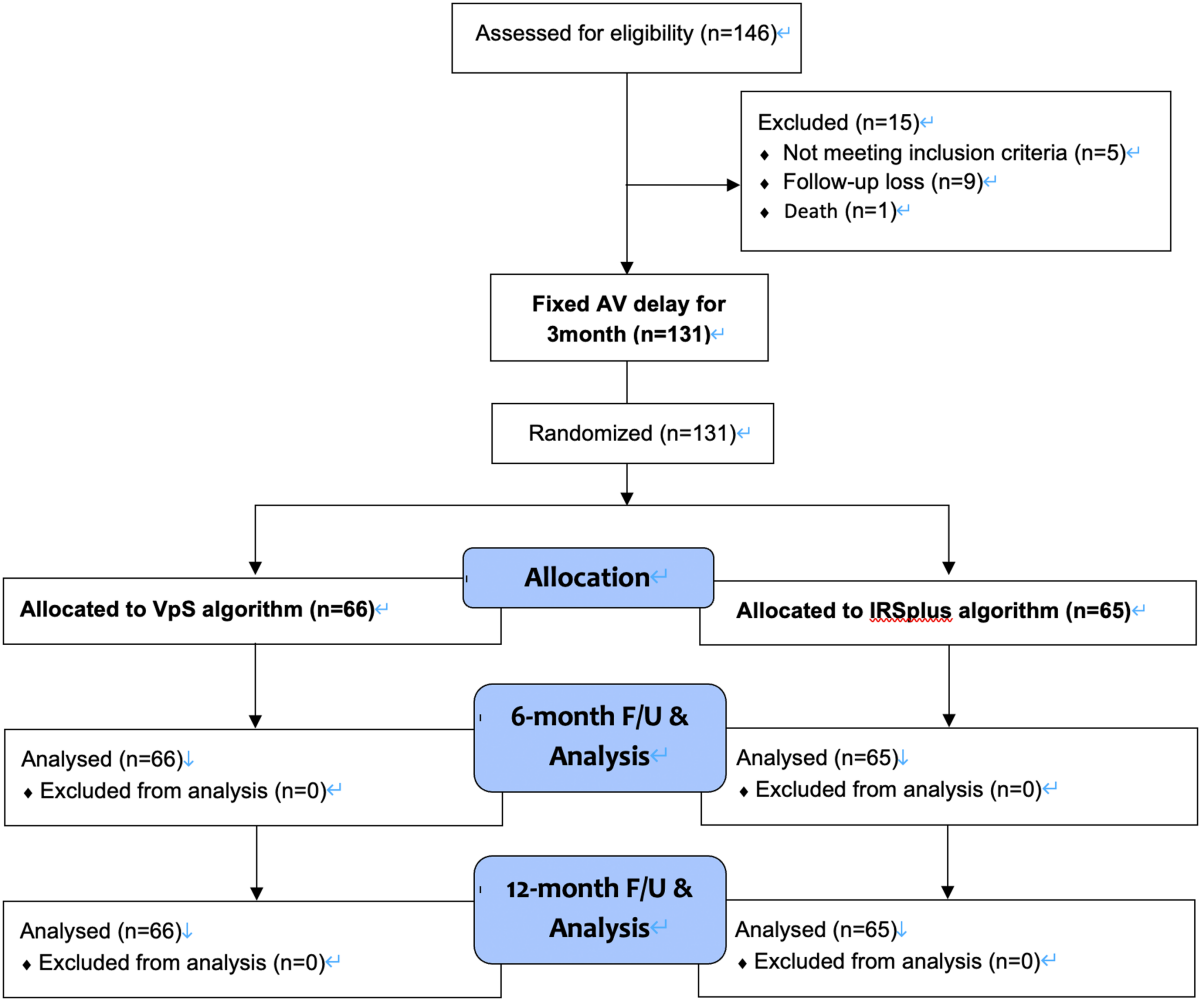
Patient enrollment and flow chart of study participants.

Figure 1 shows the design of our study. After enrollment and collection of baseline clinical characteristics, the patients' AV delay was set at a fixed interval for 3 months. The sensed AV delay was configured to the measured paced AV delay, and the programmed paced AV delay was set to 30 msec longer than this.

1. If P‐waves were observed during the procedure: intrinsic AV conduction time = As ~ Vs interval in the marker channel
  - Sensed AV delay = intrinsic AV conduction time + 20 msec
  - Paced AV delay = sensed AV delay +30 msec
2. If P‐waves were not observed during the procedure: intrinsic AV conduction time = Ap ~ Vs interval in the marker channel
  - Paced AV delay = intrinsic AV conduction time + 20 msec
  - Sensed AV delay = paced AV delay − 30 msec

Subsequently, patients were randomized 1:1 and assigned to either the VpS or IRSplus algorithm group for the following 6 months. Then, an additional 6‐month follow‐up with the same algorithm was conducted to obtain longer‐term data for evaluating the algorithms' efficacy and safety. Thorough device interrogations were performed at enrollment, at 3 months, 9 months, and at the last visit (15 months).

### 2.3 Study outcomes

The primary outcome was the ventricular pacing percentage (Vp%) at each follow‐up visit; 3‐month, 9‐month, and 15‐month. The primary outcome between the fixed AV delay group and ventricular pacing minimization algorithms group was compared. The secondary outcome was the occurrence of HF hospitalization and AF during the study period as well as the burden of atrial high‐rate episodes. The secondary outcomes during the study period were investigated. The safety outcomes included major adverse cardiac/cerebrovascular events such as myocardial infarction or stroke. A serious adverse event was defined as any medical event that resulted in death, was life‐threatening, required hospitalization, or caused substantial disability or incapacity. These were also assessed during the study period.

### 2.4 Mechanism of VpS and IRSplus algorithm

**FIGURE 2:**
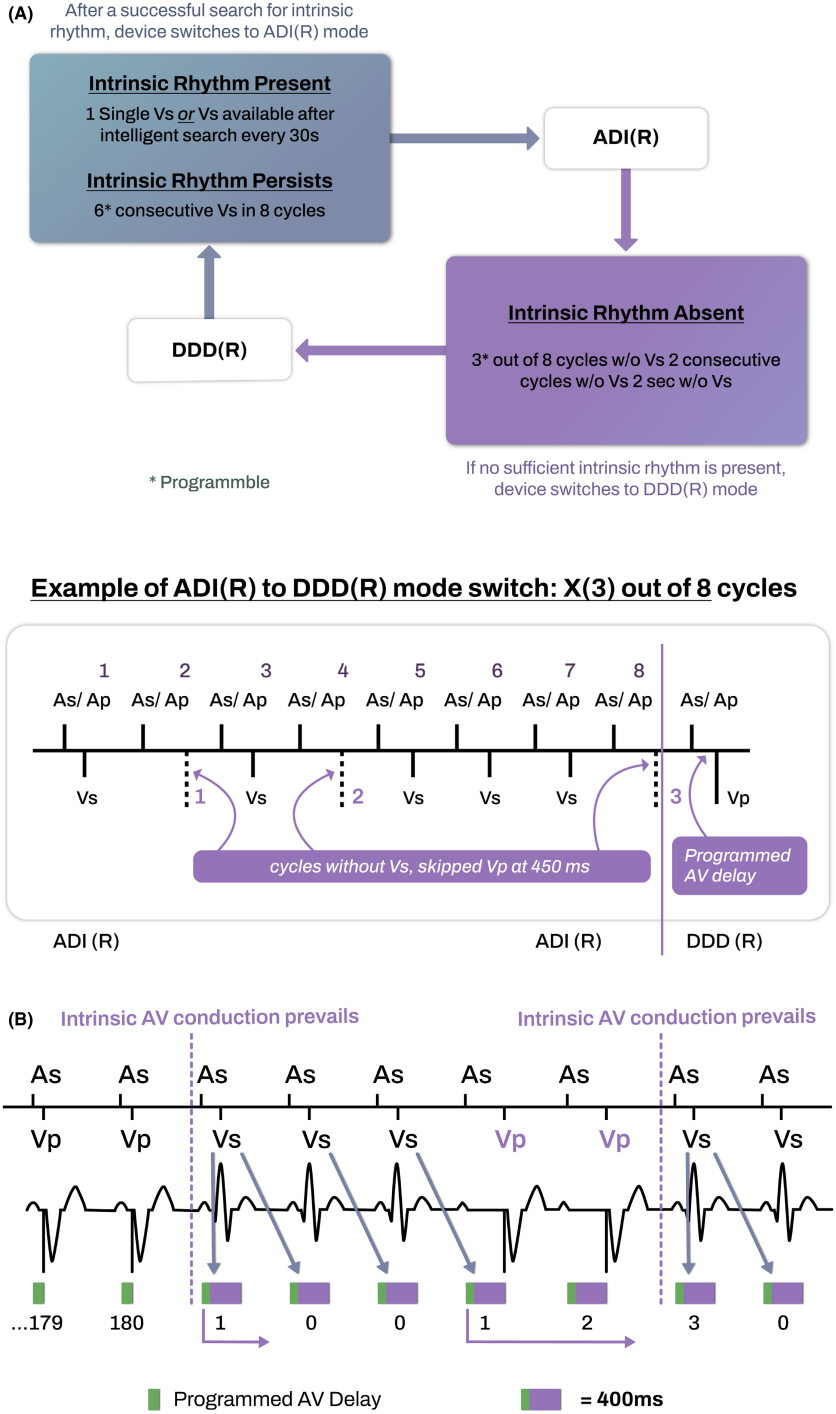
Illustration for the mechanism of ventricular pacing minimization algorithm. (A) Ventricular pace Suppression algorithm (VpS) based on ADI‐DDD mode switch (B) Intrinsic Rhythm Support (IRS) plus algorithm (IRSplus) based on atrioventricular hysteresis. See text for a more detailed explanation.

The VpS algorithm transitions between dual‐ and single‐chamber atrial modes based on the successful completion of predefined conduction assessments. This algorithm alternates between ADI(R) mode, which encourages natural AV conduction without regard to the PR interval, and DDD(R) mode, where it administers ventricular pacing at a preset AV delay. While in DDD(R) mode, the system methodically examines the AV delay, extending up to 450 ms, to ascertain the presence of intrinsic ventricular activity. This assessment is initiated under one of two scenarios: either the detection of a single spontaneous ventricular event or after continuous ventricular pacing during an incrementally expanding timeframe, starting from 30 s and extending up to 20 h after each unsuccessful search. To mitigate incessant mode toggling, the algorithm incorporates an additional conduction stability check, permitting a switch to ADI(R) mode only if a PR interval shorter than 450 ms accompanies 2–8 (adjusted to 6 for the study) of the last 8 ventricular events. Reversion to DDD(R) mode is contingent upon one of four distinct criteria: an absence of ventricular sensed events for up to 2 s; two successive cycles devoid of spontaneous events; 1–5 (3 during the study) out of 8 cycles lacking spontaneous events; or an excess of 15 transitions to DDD(R) mode per hour over a 24‐hour span (Figure 2A).

On the other hand, the IRSplus feature is designed to optimize spontaneous AV conduction, functioning through a synergy of AV hysteresis and intrinsic conduction search mechanisms. Physicians can set both sensed and paced AV intervals within a spectrum ranging from 15 to 350 ms, adjustable in 5‐ms increments across six distinct rate ranges. Activation of the AV hysteresis feature leads to an automatic extension of the AV delay to a maximum of 400 ms following any intrinsic ventricular event. Should intrinsic conduction be interrupted, the device persists with the elongated AV delay for a sequence of up to 10 cycles (reduced to 5 for the purposes of this study) before reverting to the pre‐established AV delay, fostering the potential resumption of natural conduction. Moreover, the device intermittently implements up to 10 prolonged AV delays after every 180 consecutive pacing cycles, in an effort to detect any spontaneous ventricular activity (Figure 2B).

### 2.5 Statistical analysis

Under the assumption of the Vp% of 16.7% in the fixed AV delay method and 3% in the VpS mode, with a standard deviation of 28%,^7^, ^8^, ^9^ the required number of subjects for the comparison of means between the two groups is 66 when the power is 80%, and the significance level is 0.05. Considering the dropout rate of 10%, the number of patients required to achieve the purpose of this study is 146 (73 in each group).

Randomization was performed by an independent statistician at a ratio of 1:1. Our study was a randomized pilot study, and several exploratory data analyses were done. Continuous variables are expressed as the mean value ± standard deviation or interquartile range when the values do not follow a normal distribution. Categorical variables are expressed as numbers and percentages. The paired/independent sample *t*‐test and chi‐square test were used for continuous and categorical variables if normality was accepted. If the sample did not meet the normality assumption, the following method was used: the Mann–Whitney test was used to compare within‐group continuous variables before and after the intervention, and the Wilcoxon signed‐rank test was used to analyze the differences and changes in values between the two groups. Statistical analyses were performed using the MedCalc Statistical Software (MedCalc® Statistical Software version 22.013, MedCalc Software Ltd, Ostend, Belgium). A *p*‐value of <.05 was considered statistically significant.

## 3 RESULTS

### 3.1 Patient characteristics

A total of 146 patients were assessed for eligibility, among whom 5 did not meet the inclusion criteria, 9 were lost to follow‐up, and 1 patient died. Therefore, 131 patients were finally enrolled and completed the trial protocol (Figure 1). Of these, 43 patients were male (32.8%), and the average age was 70.0 ± 8.3 years. Atrial leads were placed at the right atrial appendage in all patients. The baseline mean sensed AV delay (SAVD) on the marker channel was 190.1 ± 54.2 msec, and the mean paced AV delay (PAVD) on the marker channel was 265.4 ± 45.7 msec. Other baseline characteristics of patients are shown in Table 1.

**TABLE 1:** Baseline characteristics and mean Vp% after 3 months of fixed AV delay.

Characteristic	Total (*n* = 131)	VpS group (*n* = 66)	IRSplus group (*n* = 65)	*p*‐value for two groups
Male	43 (32.8)	22 (33.3)	21 (32.3)	.91
Age (years)	70.0 ± 8.3	69.8 ± 8.4	70.2 ± 8.3	.74
Hypertension	98 (74.8)	45 (68.2)	44 (67.7)	.95
Diabetes mellitus	45 (34.6)	14 (21.2)	13 (20.0)	.87
LA dimension (mm)	45.6 ± 14.3			
Sensed AV delay (msec)	190.1 ± 54.2	189.7 ± 53.9	190.5 ± 54.6	0.94
Paced AV delay (msec)	265.4 ± 45.7	264.8 ± 46.0	266.0 ± 45.5	.87
Programmed sensed AV delay (msec)	211.5 ± 41.6	210.1 ± 38.7	211.3 ± 42.6	.76
Programmed paced AV delay (msec)	297.9 ± 42.9	298.5 ± 40.2	297.0 ± 43.6	.81
Mean Vp% after 3‐month (%)	14.1 ± 19.4	16.9 ± 21.3	11.4 ± 17.0	.08

Abbreviations: AV, atrioventricular; LA, left atrium; Vp%, ventricular pacing percentage.

Values are presented as *n* (%) or mean ± SD.

### 3.2 Fixed AV delay versus ventricular pacing minimization algorithms

In the initial 3 months, the AV delay was set to a fixed interval based on each patient's sensed/paced AV interval, as previously detailed. The mean programmed SAVD was 211.5 ± 41.6 msec, and the mean programmed PAVD was 297.9 ± 42.9 msec.

**FIGURE 3:**
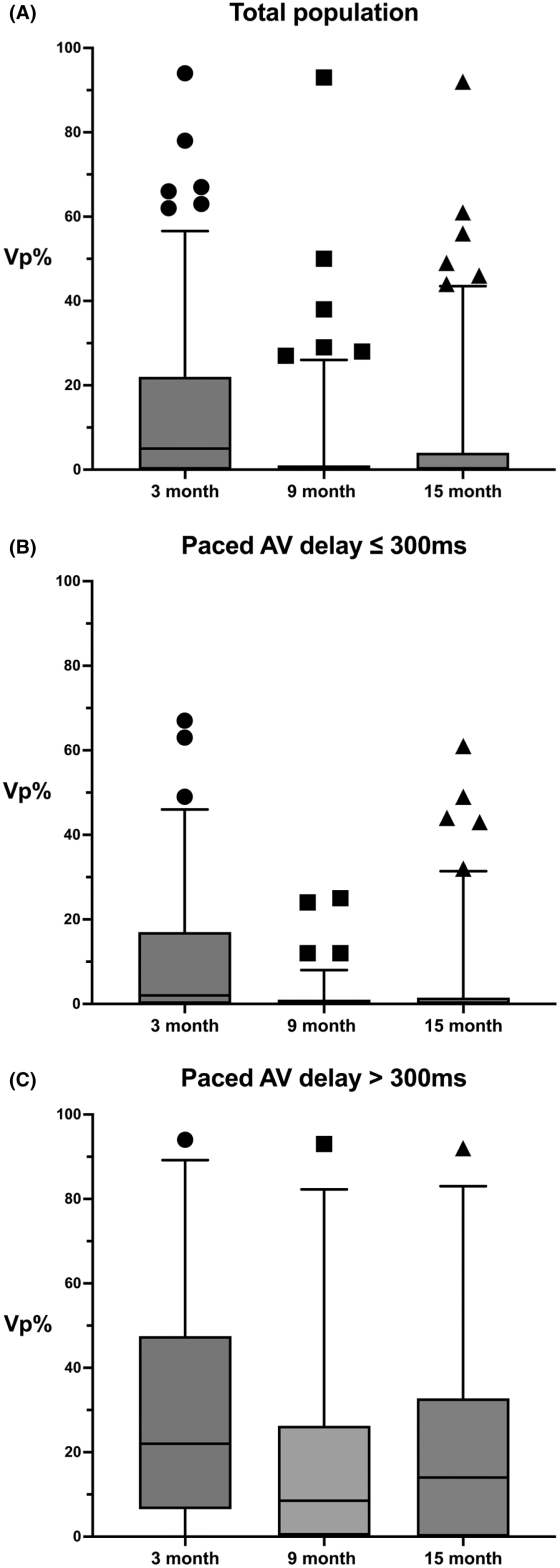
Box and whiskers plots of ventricular pacing percentage (Vp%) during the study period. The pacemaker's atrioventricular delay was fixed at programmed intervals for the first 3 months. After that, the ventricular pacing minimization algorithm was introduced at 6 months (9 months) and 12 months (15 months). Upper and lower box lines denote 75th and 25th percentiles, and the median is displayed in horizontal lines within boxes. The whiskers denote 5%–95% percentiles, while the individual data points (circles, squares, and triangles) indicate outliers. (A) Vp% of the total population at three time points: The adoption of algorithms resulted in a significant reduction of Vp%. (B) The Vp% for patients with a paced AV delay (PAVD) of 300 msec or less, whereas (C) shows the Vp% for patients with a paced AV delay greater than 300 msec, observed at 3, 9, and 15 months. In patients with PAVD ≤300 ms, the Vp% was lower than in the total population, significantly decreased after the application of the algorithm, and remained stable over time. In contrast, for the patient group with PAVD >300 ms, Vp% was quite high compared with the total population. Although there was a meaningful reduction after implementing the algorithm, the Vp% remained high and showed an increasing trend over time.

During this phase of a fixed AV delay, the mean Vp% among patients was 14.1 ± 19.4%. Following this, patients were transitioned to the phase of ventricular pacing minimization algorithms. The adoption of these algorithms resulted in a significant reduction of the mean Vp% to 4.0 ± 11.3% at the 9‐month and 6.7 ± 14.9% at 15 months (Figure 3A). Paired t‐tests for statistical analysis indicated a significant decrease in Vp% from the period of fixed AV delay to both the 9 months (mean difference 10.3 ± 15.0, *p* < .0001) and 15 months (mean difference 7.8 ± 14.3, *p* < .0001). Notably, a slight increase in Vp% was observed from the 9 months to the 15 months, which was statistically significant (mean difference 2.7, *p* = .0017).

### 3.3 Effect of paced AV delay on Vp%

To identify parameters associated with the Vp%, we analyzed the SAVD, PAVD at the time of study enrollment, and the programmed SAVD and PAVD at randomization. Correlation analysis revealed that the PAVD at the time of enrollment exhibited the highest correlation with Vp% at 9 months (correlation coefficient 0.402). Through receiver operating characteristic (ROC) curve analysis, we determined the cutoff value for PAVD capable of predicting a Vp% exceeding 5% at 9 months to be 303 msec, displaying a sensitivity of 60.9%, a specificity of 90.7%, and an area under the curve (AUC) of 0.759 (*p* < .001). Interestingly, the cutoff value for predicting a Vp% exceeding 10% was also 303 msec, with a sensitivity of 68.7%, a specificity of 88.7%, and an AUC of 0.773 (*p* = .001).

Therefore, to better investigate the efficacy of the algorithms in relation to patients' PAVD on the marker channel at the time of study enrollment, patients were stratified into two groups: those with PAVD ≤300 ms (*n* = 109) and those with PAVD >300 ms (*n* = 24). Analysis of Vp% in these groups demonstrated a pronounced effect of the algorithms in the group with PAVD ≤300 ms. Specifically, in patients with PAVD ≤300 ms, the mean Vp% during the 3‐month period with a fixed AV delay was 10.5 ± 15.7%, which significantly decreased to 1.3 ± 3.9% at 9 months (initially 6 months with the algorithm; *p* < .0001) and slightly increased to 3.6 ± 10.4% at 15 months (6–12 months with the algorithm continuation; *p* < .0001; Figure 3B). Meanwhile, in the group with PAVD >300 ms, the mean Vp% during the 3‐month fixed AV delay phase was notably higher at 30.8 ± 25.8%. With the algorithm, the mean Vp% was reduced to 15.7 ± 21.5% at 9 months (*p* = .0004), but it rebounded to 19.9 ± 22.7% at 15 month (*p* = .0022) (Figure 3C).

### 3.4 Comparison between ventricular pacing minimization algorithms: VpS versus IRSplus

**FIGURE 4:**
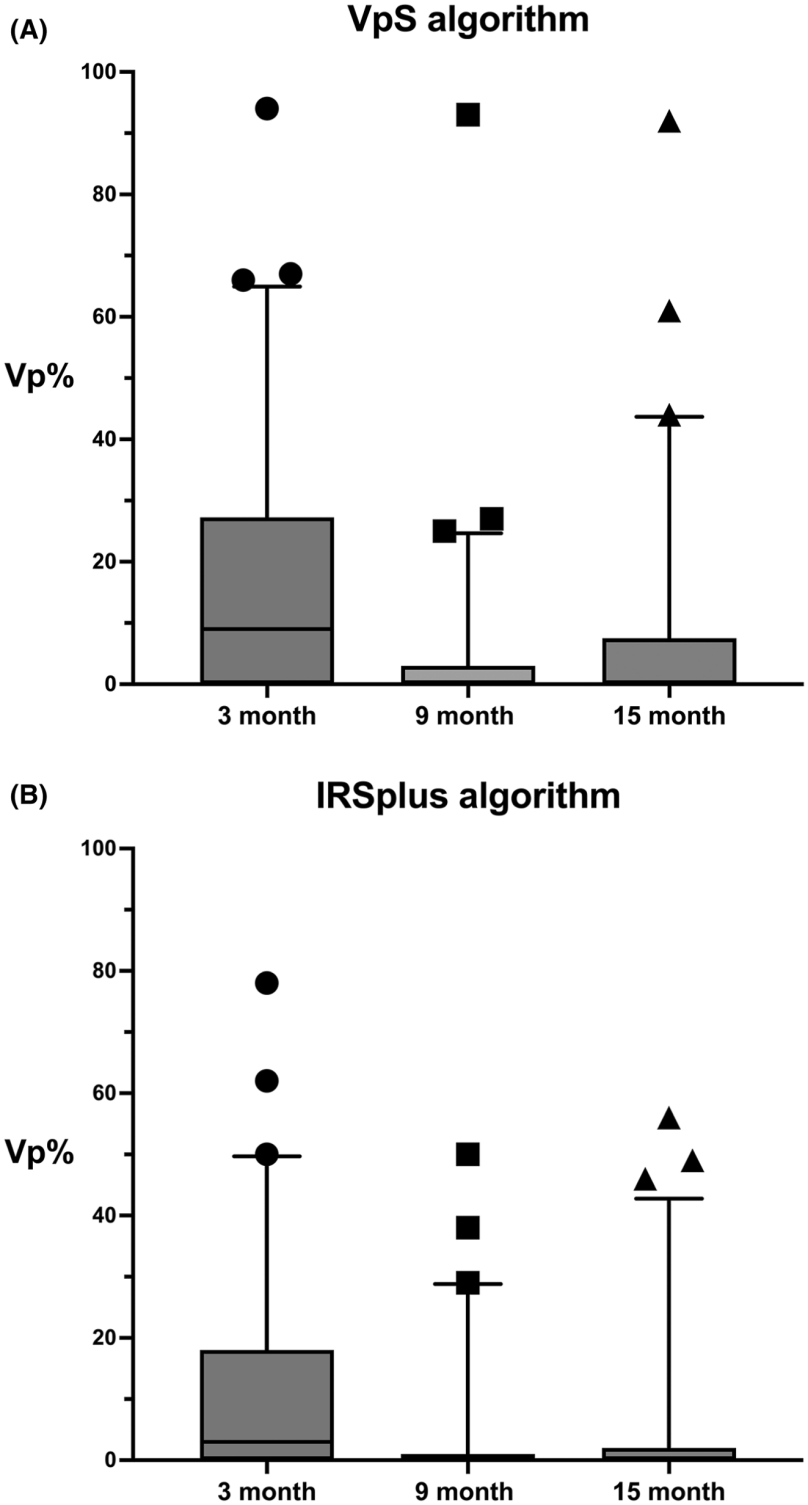
The ventricular pacing percentage (Vp%) over time using the VpS (Ventricular pace Suppression) algorithm and the IRSplus (Intrinsic Rhythm Support plus) algorithm. The pacemaker's atrioventricular delay was fixed at programmed intervals for the first 3 months. After that, the ventricular pacing minimization algorithm was introduced at 6 months (9 months) and 12 months (15 months). Upper and lower box lines denote 75th and 25th percentiles, and the median is displayed in horizontal lines within boxes. The whiskers denote 5%–95% percentiles, while the individual data points (circles, squares, and triangles) indicate outliers. (A) A trend of initial Vp% reduction with the VpS algorithm at 9 months, followed by a slight increase at 15 months. In contrast, (B), with the IRSplus algorithm, displays a more pronounced and stable reduction in Vp% at 9 months, which is maintained at 15 months. However, there were no statistical differences between the two groups (see text).

Following a 3‐month period with a fixed AV delay, patients were randomized into two groups in a 1:1 ratio: the VpS algorithm group (*n* = 66) and the IRSplus algorithm group (*n* = 65). The mean Vp% observed during the study period were as follows (Figure 4A,B):

- *VpS Group*: During 3 months of fixed AV delay: 16.9 ± 21.3%.
- At 9 months (initial 6 months with the algorithm): 4.4 ± 12.8%.
- At 15 months (6–12 months with the algorithm): 8.5 ± 17.2%.
- *IRSplus Group*: During 3 months of fixed AV delay: 11.4 ± 17.0%.
- At 9 months (initial 6 months with the algorithm): 3.6 ± 9.6%.
- At 15 months (6–12 months with the algorithm): 4.8 ± 12.0%.

The mean Vp% differences and their statistical significance when transitioning from the fixed AV delay phase to the algorithm application were as follows:

- *VpS Group*: From 3 months of fixed AV delay to the initial 6 months with the algorithm: −12.5 ± 16.2, *p* < .0001.
- From 3 months of fixed AV delay to 6–12 months with the algorithm: −8.7 ± 16.2, *p* = .0001
- From the initial 6 months with the algorithm to 6–12 months with the algorithm: 4.0 ± 10.2, *p* = .0023.
- *IRSplus Group*: From 3 months of fixed AV delay to the initial 6 months with the algorithm: −7.9 ± 13.3, *p* < .0001.
- From 3 months of fixed AV delay to 6–12 months with the algorithm: −6.8 ± 12.1, *p* < .0001.
- From the initial 6 months with the algorithm to 6–12 months with the algorithm: 1.3 ± 6.5, *p* = 0.129.

These results indicate that both algorithms successfully reduced RV pacing compared to the fixed AV delay. The VpS group demonstrated a more significant initial reduction but experienced a slight but statistically significant increase in pacing percentage during an additional 6 months of follow‐up. Meanwhile, the IRSplus group exhibited a more consistent and stable reduction in ventricular pacing over time.

Although not the primary object of our study, we conducted an explorative analysis of Vp% between the two algorithm groups (VpS and IRSplus). Due to the skewness and nonnormality observed in the distribution of mean Vp% differences, the Mann–Whitney *U* test, a nonparametric test, was employed to compare the median changes between the two groups. The analysis revealed no statistically significant differences between the two algorithms in the reduction of Vp% from the fixed AV delay phase to 6 months of algorithm use (*U*‐statistic: 1695.5, *p*‐value: .063) or from the fixed AV delay to 6–12 months of algorithm application (*U*‐statistic: 1963.5, *p*‐value: .519; Table S1).

### 3.5 Secondary and safety outcomes

During the study period, HF hospitalization and ECG‐documented clinical AF did not occur. A small number of patients showed atrial high‐rate episodes of below 5 min: 4 in the initial 3 months, 4 between 3 and 9 months, and 6 between 9 and 15 months. However, no further analysis was performed due to the small number and heterogeneity of the events among the study cohorts. In addition, the clinical significance of AHREs of 5 min or less is currently unclear. The major adverse cardiac/cerebrovascular events and serious adverse events were not observed.

## 4 DISCUSSION

In this study, we sought to evaluate the efficacy of ventricular pacing minimization algorithms—VpS and IRSplus—compared to the fixed AV delay method through a structured randomized pilot study. Our findings reveal that the VpS and IRSplus algorithms effectively and significantly reduce the Vp% compared to a fixed AV delay. The effectiveness of the algorithms was pronounced in the group with PAVD ≤300 ms. In the group with PAVD >300 ms, the use of the algorithms was effective, but the Vp% values were higher, and there was a larger increase in Vp% over time. Although the study was not designed to compare the two algorithms, explorative analysis revealed no statistical difference in the efficacy of the two algorithms.

After recognizing the detrimental effects of unnecessary RV pacing, manufacturers have developed algorithms to minimize RV pacing. These algorithms are classified into two categories: AVH algorithm versus rapid mode switch algorithm. Depending on the manufacturer, a device may incorporate both or just one of these algorithms. There have been few recent studies directly comparing the efficacy of fixed AV delay with RV pacing minimization algorithms in the same patients. In our study, the use of these algorithms significantly reduced the Vp% compared to the fixed AV delay. Therefore, algorithms are recommended to use for individuals with SND. This is clearly indicated by AHA/ACC/HRS^10^ and ESC Guidelines.^3^

Currently, there is no consensus on which algorithm is superior or which patient population might benefit more from a specific algorithm. The MVP algorithm (Medtronic mode switch algorithm) and SafeR algorithm (Sorin mode switch algorithm) have technically been shown to reduce the Vp% to negligible levels.^11^, ^12^ However, the algorithms achieved only a modest decrease in the incidence of persistent AF compared with traditional DDD pacing.^13^, ^14^ Additionally, the SavePACE trial, which demonstrated a reduction in persistent AF using Medtronic Search AV (Medtronic AVH), found that the mode switch algorithm, employed in 10% of the study participants, did not lead to a significant decrease in persistent AF compared to standard DDDR pacing.^13^ Meanwhile, the ANSWER trial indicated that the SafeR algorithm was able to significantly reduce the risk of cardiac death or hospitalization for heart failure by 51%, and lower the chances of cardiovascular hospitalizations by 30%, relative to conventional DDD pacing.^15^ However, recent meta‐analysis revealed that mode switch algorithms did not improve clinical outcomes and were not superior to standard DDD programming in reducing incidence of persistent AF, all‐cause hospitalization, or all‐cause mortality.^7^ Interestingly, a recent study that compiled data from two identical large prospective observational studies, BRADYCARE I in the US and BRADYCARE II in Europe, Africa, and Asia, found that VIP algorithm (Abbott AVH algorithm), compared with standard pacing, was associated with a lower 1‐year incidence of hospitalization for HF hospitalization and of persistent AF.^16^ The VpS and IRSplus algorithms were introduced relatively recently, and while long‐term data are limited, in our study, the IRSplus group tended to have a lower and more stable Vp% than the VpS group without statistical significance. This nuance is in line with recent studies published by Calvi et al., which found that the IRSplus was slightly more efficient than VpS in reducing VP%.^8^

The degree of degeneration in the conduction system should also be considered when devising RV pacing minimization strategies. In our study, patients with a PAVD of more than 300 msec showed significantly higher Vp% compared to those with less than 300 msec, and this trend persisted even after algorithm application. In the studies by Calvi et al., the analysis was divided based on SAVD at 170 msec and 270 msec, with patients above 270 msec showing a high degree of Vp% similar to our findings, despite algorithm use.^8^ However, an AV interval of 270–300 msec or more is considered beyond the physiological range, and it is well known that excessive prolongation of AV conduction time can result in AV decoupling (which may induce diastolic mitral regurgitation), VA coupling (reversal of the left‐sided AV timing sequence), and AV uncoupling (transient breakdown in 1:1 AV conduction).^17^ In addition, programming that allows for markedly prolonged AV delays and does not correct first‐degree AV block can worsen mitral regurgitation, shorten diastolic filling, and cause pacemaker syndrome.^18^ Therefore, the AV interval should be short enough to optimize AV synchrony, while being long enough to promote intrinsic AV conduction. According to these findings, in patients with prolonged AV conduction, AVH algorithms or mode switch algorithms limited to AV delays shorter than 250 ms may be more physiological and improve clinical outcomes.^16^ Further research is needed to ascertain whether applying pacing algorithms that allow for the maximum AV interval would be clinically beneficial in patients with SND with marked first‐degree AV block (impaired AV conduction). Additionally, our study observed a slight increase in the Vp% over time, which underscores the importance of considering the progressive nature of conduction system degeneration. Recently emerged conduction system pacing, especially left bundle branch area pacing, is expected to be a good solution to this issue.

Our study has several limitations. Despite randomization, the sample size of our study was relatively small, and the study was conducted solely in South Korea, which may limit the external validity of the results. The follow‐up period was limited to 15 months. Longer follow‐up durations are necessary to fully understand the long‐term effects and sustainability of the ventricular pacing suppression algorithms. Depending on the site of insertion of the pacemaker lead, the AV conduction time perceived by the pacemaker may vary significantly. For example, it is known that pacing from the low atrial septum shortens the PAVD. In our patients, all of the atrial leads were placed at the right atrial appendage, which might have influenced the study results.

## 5 CONCLUSION

Even when set to an AV delay sufficiently longer than the patient's intrinsic (laboratory confirmed) SAVD/PAVD, the fixed AV delay method showed a meaningfully higher percentage of ventricular pacing than ventricular pacing minimization algorithms, VpS and IRSplus. Therefore, the application of the algorithm is strongly recommended in SND patients with intact AV conduction. However, in patients with a prolonged AV interval (such as those with a PAVD of 300 or more, as in our study), high Vp% may occur even with algorithm use, and it is important to consider the potential for progressive degeneration of the conduction system over time. In this group of patients, establishing additional strategies, such as algorithms limited to AV delays shorter than the physiological range or conduction system pacing, may be necessary to achieve more physiological pacing.

## AUTHOR CONTRIBUTIONS

Conceptualization: Seongwook Han; Formal analysis: Jongmin Hwang; Investigation: Hyoung‐Seob Park, Tae‐Wan Chung, Minsu Jung, Seung‐Jung Park, Chan‐Hee Lee, Jin Hee Ahn, Eue‐Keun Choi, Myung Hwan Bae, Young Soo Lee, Sang Won Park, Dae In Lee, Yoo‐Ri Kim, Min‐Soo Ahn, Jaemin Shim; Writing—original draft: Jongmin Hwang; Writing—review and editing: Seongwook Han.

## FUNDING INFORMATION

This study was sponsored by BIOTRONIK SE & Co. KG.

## CONFLICT OF INTEREST STATEMENT

Authors declare no conflict of interests for this article.

## PATIENT CONSENT STATEMENT

All the patients provided written informed consent before enrollment.

## CLINICAL TRIAL REGISTRATION

This trial was registered with ClinicalTrials.gov, number NCT 03843242 (LEADER (EvaLuation of ventricular pacing suppression Algorithms in Dual chamber pacEmakeR) study).

## Supporting information


Table S1.

## Data Availability

The data sets generated during and/or analyzed during the current study are available from the corresponding author upon reasonable request. The study protocol was approved by the Research Ethics Committee of Keimyung University Dongsan Medical Center (DSMC 2018‐08‐0222). Then, the ethics committees and corresponding health authorities of all study participant sites approved the protocol.

## References

[1] Lamas GA , Lee KL , Sweeney MO , Silverman R , Leon A , Yee R , et al. Ventricular pacing or dual‐chamber pacing for sinus‐node dysfunction. N Engl J Med. 2002;346:1854–1862. 10.1056/NEJMoa013040 12063369

[2] Nielsen JC , Thomsen PE , Højberg S , Møller M , Vesterlund T , Dalsgaard D , et al. A comparison of single‐lead atrial pacing with dual‐chamber pacing in sick sinus syndrome. Eur Heart J. 2011;32:686–696. 10.1093/eurheartj/ehr022 21300730

[3] Glikson M , Nielsen JC , Kronborg MB , Michowitz Y , Auricchio A , Barbash IM , et al. 2021 ESC guidelines on cardiac pacing and cardiac resynchronization therapy. Eur Heart J. 2021;42:3427–3520. 10.1093/eurheartj/ehab364 34586378

[4] Wilkoff BL , Cook JR , Epstein AE , Greene HL , Hallstrom AP , Hsia H , et al. Dual‐chamber pacing or ventricular backup pacing in patients with an implantable defibrillator: the dual chamber and VVI implantable defibrillator (DAVID) trial. JAMA. 2002;288:3115–3123. 10.1001/jama.288.24.3115 12495391

[5] Sweeney MO , Hellkamp AS , Ellenbogen KA , Greenspon AJ , Freedman RA , Lee KL , et al. Adverse effect of ventricular pacing on heart failure and atrial fibrillation among patients with normal baseline QRS duration in a clinical trial of pacemaker therapy for sinus node dysfunction. Circulation. 2003;107:2932–2937. 10.1161/01.CIR.0000072769.17295.B1 12782566

[6] Schuger CD , Singh G . Tying ourselves in knots to avoid ventricular pacing in sick sinus syndrome: does it matter? JACC Clin Electrophysiol. 2017;3:491–493. 10.1016/j.jacep.2017.01.003 29759605

[7] Shurrab M , Healey JS , Haj‐Yahia S , Kaoutskaia A , Boriani G , Carrizo A , et al. Reduction in unnecessary ventricular pacing fails to affect hard clinical outcomes in patients with preserved left ventricular function: a meta‐analysis. Europace. 2017;19:282–288. 10.1093/europace/euw221 28175255

[8] Calvi V , Pisanò EC , Brieda M , Melissano D , Castaldi B , Guastaferro C , et al. Atrioventricular interval extension is highly efficient in preventing unnecessary right ventricular pacing in sinus node disease. JACC: Clin Electrophysiol. 2017;3:482–490. 10.1016/j.jacep.2016.11.011 29759604

[9] Davy JM , Hoffmann E , Frey A , Jocham K , Rossi S , Dupuis JM , et al. Near elimination of ventricular pacing in SafeR mode compared to DDD modes: a randomized study of 422 patients. Pacing Clin Electrophysiol. 2012;35:392–402. 10.1111/j.1540-8159.2011.03314.x 22309303

[10] Kusumoto FM , Schoenfeld MH , Barrett C , Edgerton JR , Ellenbogen KA , Gold MR , et al. 2018 ACC/AHA/HRS guideline on the evaluation and management of patients with Bradycardia and Cardiac Conduction Delay: a report of the American College of Cardiology/American Heart Association Task Force on Clinical Practice Guidelines and the Heart Rhythm Society. Circulation. 2019;140:e382–e482. 10.1161/CIR.0000000000000628 30586772

[11] Chen S , Chen K , Tao Q , Zheng L , Shen F , Wu S , et al. Reduction of unnecessary right ventricular pacing by managed ventricular pacing and search AV+ algorithms in pacemaker patients: 12‐month follow‐up results of a randomized study. Europace. 2014;16:1595–1602. 10.1093/europace/euu055 24706091

[12] Thibault B , Ducharme A , Baranchuk A , Dubuc M , Dyrda K , Guerra PG , et al. Very low ventricular pacing rates can be achieved safely in a heterogeneous pacemaker population and provide clinical benefits: the CANadian multi‐centre randomised study‐spontaneous AtrioVEntricular conduction pReservation (CAN‐SAVE R) trial. J Am Heart Assoc. 2015;4:e001983. 10.1161/JAHA.115.001983 26206737 PMC4608083

[13] Sweeney MO , Bank AJ , Nsah E , Koullick M , Zeng QC , Hettrick D , et al. Minimizing ventricular pacing to reduce atrial fibrillation in sinus‐node disease. N Engl J Med. 2007;357:1000–1008. 10.1056/NEJMoa071880 17804844

[14] Boriani G , Tukkie R , Manolis AS , Mont L , Pürerfellner H , Santini M , et al. Atrial antitachycardia pacing and managed ventricular pacing in bradycardia patients with paroxysmal or persistent atrial tachyarrhythmias: the MINERVA randomized multicentre international trial. Eur Heart J. 2014;35:2352–2362. 10.1093/eurheartj/ehu165 24771721 PMC4163193

[15] Stockburger M , Boveda S , Moreno J , da Costa A , Hatala R , Brachmann J , et al. Long‐term clinical effects of ventricular pacing reduction with a changeover mode to minimize ventricular pacing in a general pacemaker population. Eur Heart J. 2015;36:151–157. 10.1093/eurheartj/ehu336 25179761 PMC4297468

[16] Arnold M , Richards M , D'Onofrio A , Faulknier B , Gulizia M , Thakur R , et al. Avoiding unnecessary ventricular pacing is associated with reduced incidence of heart failure hospitalizations and persistent atrial fibrillation in pacemaker patients. Europace. 2023;25:euad065. 10.1093/europace/euad065 36942949 PMC10227662

[17] Sweeney MO , Ellenbogen KA , Tang AS , Johnson J , Belk P , Sheldon T . Severe atrioventricular decoupling, uncoupling, and ventriculoatrial coupling during enhanced atrial pacing: incidence, mechanisms, and implications for minimizing right ventricular pacing in ICD patients. J Cardiovasc Electrophysiol. 2008;19:1175–1180. 10.1111/j.1540-8167.2008.01226.x 18554192

[18] Barold SS , Herweg B . Conventional and biventricular pacing in patients with first‐degree atrioventricular block. Europace. 2012;14:1414–1419. 10.1093/europace/eus089 22516061

