# Supplementary material for: Evaluation of ventricular pacing suppression algorithms in dual chamber pacemaker: Results of “LEADER” study
Source: J Arrhythm. 2024 Jul 16;40(4):965–74. doi: 10.1002/joa3.13117 (PMC11317712; doi:10.1002/joa3.13117)
Supplement: Supplementary file 1 — Table S1. [file JOA3-40-965-s001.docx]

**Supplementary Table.** Vp% Comparison between the fixed AV Delay phase and ventricular pacing minimization algorithm usage phase

| **Characteristic** | **VpS Group**  **(n=66)** | **IRSplus Group**  **(n=65)** | **p-value** |
| --- | --- | --- | --- |
| Mean Vp% of fixed AV delay | 16.9 ± 21.3 | 11.4 ± 17.0 | 0.217 |
| Mean Vp% during 6month of algorithm use | 4.4 ± 12.76 | 3.6 ± 9.56 |  |
| Mean Vp% during 6-12month of algorithm use | 8.5 ± 17.18 | 4.8 ± 11.98 |  |
| Median Vp% of fixed AV delay | 9.0 [0.0, 26.25] | 3.0 [0.0, 18.0] |  |
| Median Vp% during 6month of algorithm use | 0.0 [0.0, 2.75] | 0.0 [0.0, 1.0] |  |
| Median Vp% during 6-12month of algorithm use | 0.0 [0.0, 7.0] | 0.0 [0.0, 2.0] |  |
| Mean Reduction (3m to 9m) | 12.7 | 7.9 |  |
| Median Reduction (3m to 9m) | 19.0 | 13.0 | 0.063 |
| Mean Reduction (3m to 15m) | 8.7 | 6.6 |  |
| Median Reduction (3m to 15m) | 15.0 | 13.0 | 0.519 |

* Values are presented as n (%) or mean ± SD. Vp%: ventricular pacing percentage; LA: left atrium; AV: atrioventricular.
